# Identification and Characterization of Two Novel RNA Editing Sites in *grin1b* Transcripts of Embryonic *Danio rerio*

**DOI:** 10.1155/2012/173728

**Published:** 2012-02-27

**Authors:** Pedro Pozo, Barry Hoopengardner

**Affiliations:** Department of Biomolecular Sciences, Central Connecticut State University, 1615 Stanley Street, New Britain, CT 06050, USA

## Abstract

Discovering RNA editing sites in model organisms provides an insight into their adaptations in addition to finding potential sites for the regulation of neural activity and the basis of integrated models of metazoan editing with a variety of applications, including potential clinical treatments of neural dysregulation. The zebrafish, *Danio rerio*, is an important vertebrate model system. We focused on the *grin1b* gene of zebrafish due to its important function in the nervous tissue as a glutamate receptor. Using a comparative sequence-based approach, we located possible RNA editing events within the *grin1b* transcript. Surprisingly, sequence analysis also revealed a new editing site which was not predicted by the comparative approach. We here report the discovery of two novel RNA editing events in *grin1b* transcripts of embryonic zebrafish. The frequency of these editing events and their locations within the *grin1b* transcript are also described.

## 1. Introduction

 Adenosine-to-inosine RNA editing occurs primarily in components of neural function and synaptic transmission; adenosines within the targeted transcript are converted to inosine which is interpreted as guanosine during translation and manifests as A/G mixed signals in sequence chromatograms. Several editing sites have been reported in zebrafish (*Danio rerio*) including one located in the *GRIA2* gene [1]. There are several NMDA receptors coded in the zebrafish genome [2], and we have discovered editing in a member of this group, *grin1b *(*NMDAR1.2*).

 Grin1b is an ionotropic NMDA glutamate receptor located on zebrafish chromosome 5. The coding sequence of the mature *grin1b* transcript is 2,814 nucleotides. Translation of this mature mRNA produces a protein product made up of 937 amino acids and serves as a postsynaptic glutamate receptor and ligand-gated ion channel.

 NMDA receptors are important in neural plasticity and long-term potentiation; hyperexcitation of the receptors can lead to neuronal death. Understanding the factors that influence the regulation of these receptors is therefore important for the treatment of a variety of human neurological disorders.

## 2. Materials

### 2.1. RNA Isolation and RT-PCR

RNA was extracted from wild-type *Danio rerio* EK strain embryos 60–72 hours old using the TRI reagent protocol (MRC, Cincinnati, OH); the nomenclature of the editing sites incorporated an “E” designation to emphasize that embryonic tissue was used. All Danio research was done in accordance with institutional IACUC guidelines.

 Reverse transcription was performed using Invitrogen MMLV-RT and associated components (Carlsbad, CA), primed with a polythymidylate (polyT) primer. Subsequent PCR was performed using Promega GoTaq (Madison, WI) and specific primer sets (see Tables 1 and 2) from IDT (Coralville, IA). DNA for genomic controls was isolated using the Qiagen (Valencia, CA) DNA mini procedure from the same tissue sample set used for RNA.

### 2.2. Sequencing and Restriction Digests

 Electrophoresis was done on 1.2% gels and gel extraction as per Qiagen (Valencia, CA) gel extraction kit instructions. 

Sequencing services were provided by SeqWright (Fisher, Houston, TX). Restriction digests were performed with NEB (Ipswich, MA) enzymes *MluI* and *BstNI*, following NEB recommendations regarding temperature and inclusion of bovine serum albumin (BSA).

 Intensity values based on ethidium bromide fluorescence were acquired using Kodak Gel Logic (Rochester, NY) software and adjustments as described in the text.

## 3. Results

**Figure 1:**
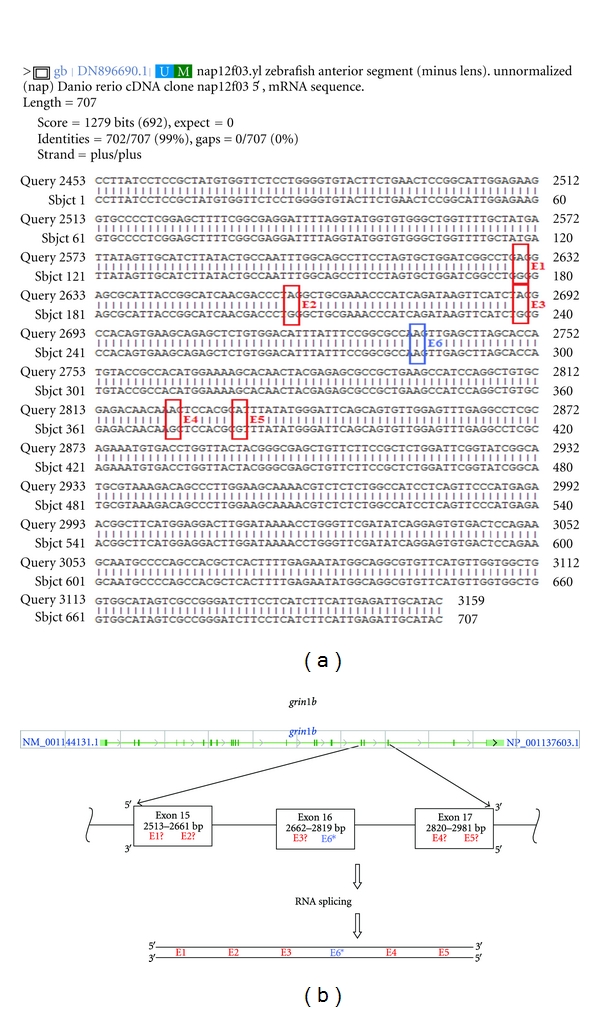
Alignments. A schematic showing the relative (a) and genomic (b) locations of the 5 initially predicted RNA editing events obtained from BLAST searches within the *grin1b* transcript. Predicted sites E1–E5 are located in a stretch of about 468 bases in the mature mRNA corresponding to exons 15 through 17 (as per zfin.org, ensembl.org). Schematic corresponds to NCBI representation of the *grin1b* gene in 2011. *The E6 site was discovered only after direct sequencing.

 A BLAST search was performed using *grin1b* coding sequence (CDS) against NCBI databases; substantial regions of sequence identity were detected only against Danio sequences. Danio *grin1b* CDS was then compared against EST sequences, and upon filtering though several hits we selected a sequence comparison containing several A–G mismatches (Figure 1). These A–G mismatches were interpreted as potential A-to-I RNA editing sites.

 The sequences of interest selected in this study involved RNA : RNA comparisons of curated zebrafish *grin1b* transcripts, many from the anterior segment of the eye and possibly encompassing the nervous tissue of the retina (Figure 1) (see also [2]: NR1.2 expression pattern). As a result, the sequences compared aligned perfectly to one another except for five A-G mismatched positions, hereafter referred to as E1, E2, E3, E4, and E5 (Figure 1, Section 2). This predictive result was encouraging and prompted us to continue our research using the sites identified by this BLAST search.

 We mapped editing sites E1 and E2 to *grin1b* exon 15, E3 to exon 16, and E4 and E5 to exon 17 (Figure 1(b), Table 1) (as per zfin.org, ensembl.org). As shown in Figure 1, all five candidate editing sites were located in adjacent exons within a 468 nucleotide region in the mature *grin1b* mRNA.

**Figure 2:**
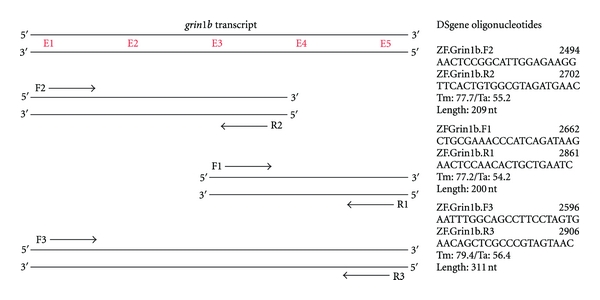
Editing locations. Using the DS Gene computer program (Accelrys), primer pairs were designed to amplify the *grin1b* transcript regions containing the predictive editing events. A total of three primer pairs were constructed, two of which (F1/R1 and F2/R2) amplified a region containing at least three of the five possible edits, and one primer pair (F3/R3) targeted to amplify a region containing all five possible edits.

**Figure 3:**
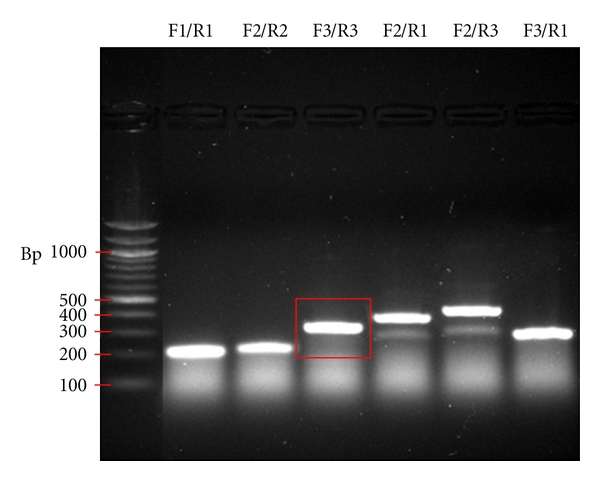
F3/R3 product. RT-PCR products (Invitrogen MMLV-RT, GoTaq Green Polymerase). Several reactions successfully produced robust bands at the predicted sizes: 200 and 209 nucleotides for F1/R1 and F2/R2, respectively, and 311 nucleotides for F3/R3. Since the product of primer pair F3/R3 (outlined in a red box) encompassed all 6 possible editing sites, this RT-PCR band product was extracted for sequencing (100 bp ladder, New England Biolabs, Ipswich, MA).

 We were then able to design oligonucleotides aimed at amplifying the regions of the *grin1b* transcript containing the potential editing sites using Accelrys DS Gene software. Primer pairs (Figure 2) were chosen for subsequent RT-PCR reactions. The primer combination Forward3/Reverse3 (F3/R3) produced a strong band at 311 nucleotides (Figure 3); this product represented a region encompassing all five putative editing events (Figure 3) and was extracted for further sequence analysis of the predicted RNA editing sites within the *grin1b* transcript.

**Figure 4:**
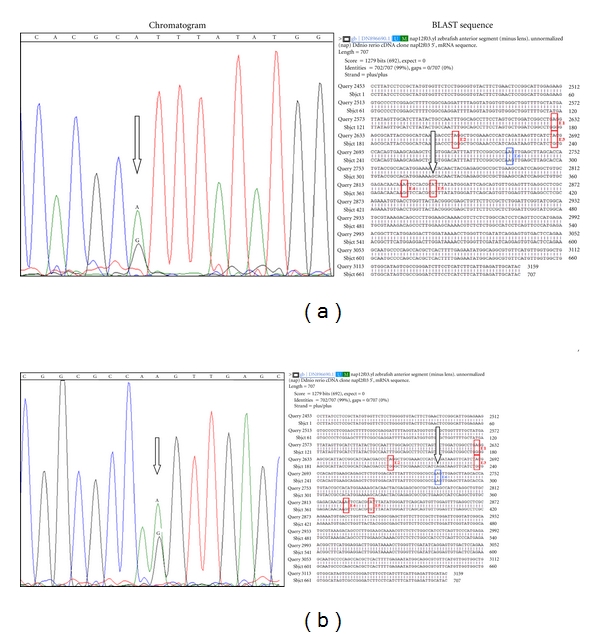
Sequencing results; E5, E6. Chromatogram sequence analysis of the RT-PCR product obtained using the F3/R3 primer pairs. The sequences show double peaks representing A/G mixed signals corresponding to position E5 ((a) open black arrow) and to a new position, E6 ((b) open black arrow and outlined in blue), in the BLAST sequence.

 Upon sequencing, the extracted product showed no chromatographic evidence of A/G mixed signals for sites E1, E2, E3, and E4; however, the predicted site E5 showed a double peak corresponding to an A/G mixed signal (Figure 4(a)). Surprisingly, upon further analysis of the chromatogram sequence, we were able to detect another A/G mixed signal which we named E6 (based on the chronology of our predictions and not transcript position) (Figure 4(b)), although E6 did not show up as an A–G mismatch in the original BLAST comparisons. This result illustrates the limitations of comparative approaches in editing site predictions. We interpret the false positives in the initial screen as rare single nucleotide polymorphisms or polymerase errors in cloned transcripts.

 We proceeded to replicate our results by conducting two separate, additional RT-PCR reactions (combined total of 3 independent RT-PCR reactions) under the same conditions to verify the occurrence of these editing sites. As expected, these produced robust bands at the size predicted and confirmed A/G mixed signals corresponding to E5 and E6 in both new reactions (data not shown).

**Figure 5:**
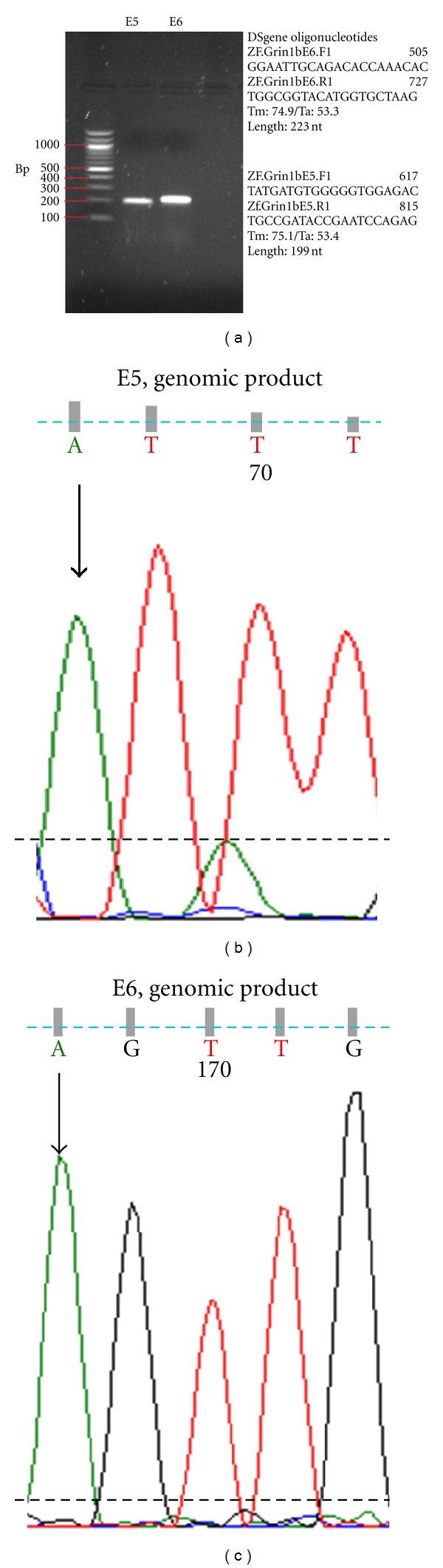
Genomic products; E5, E6. PCR amplification of regions within the *grin1b* gene corresponding to E5 and E6 potential RNA editing sites. As predicted by the DS Gene computer program (Accelrys), primers designed to target the genomic E5 sequence (ZF.Grin1bE5.F1 and ZF.Grin1bE5.R1) produced a strong band at around 200 nucleotides. Primers aimed at amplifying the genomic E6 sequence (ZF.Grin1bE6.F1 and ZF.Grin1bE6.R1) produced a strong band at around 220 nucleotides. Both of these bands were extracted for sequencing reactions. Amplicons from genomic DNA show no evidence of A/G polymorphism (A only). Dashed black lines represent noise levels per sample.

 The next step in our investigation was aimed at confirming these signals as editing sites, rather than single nucleotide polymorphisms, in the *grin1b* transcript of zebrafish. For this purpose we amplified the genomic region of *grin1b* in the region of our candidates E5 and E6. Since single nucleotide polymorphisms (SNPs) can often be misinterpreted as RNA editing events, it was necessary to amplify and sequence the genomic *grin1b* regions to distinguish between these explanations for the mixed chromatographic signals; ADAR editing enzymes do not edit DNA. Therefore, we designed two new sets of primers for subsequent zebrafish PCR reactions using genomic DNA; these primers included regions from the introns bracketing the regions corresponding to E5 and E6, separately, and do not generate products when used in RT-PCR (data not shown). We conducted separate PCR reactions for E5 and E6.  Figure 5 shows the results of these PCR reactions where two distinct bands are visible at around 200 base pairs (bp) and 223 bp for E5 and E6, respectively; after sequencing, only adenosine signal (no detectable guanosine above background, later confirmed by restriction digests) was observed at either site (Figure 5).

 We chose to use restriction digestion and densitometry to quantitate levels of RNA editing. Editing occurs within a transcript population and may result in the creation or destruction of a restriction enzyme site. The unedited and edited transcript forms were analyzed using New England Biolabs (NEB) and Accelrys DS Gene software, and restriction enzymes were chosen that could differentiate between editing and lack of editing in a transcript at either site 5 or site 6, separately. *MluI* (A/CGCGT) was chosen for site 5, and *BstNI* (CC/WGG) was chosen for site 6. Editing at site 5 creates an *MluI* restriction enzyme site (ACGC**A**T to A/CGC**G**T), while editing at site 6 creates a *BstNI* restriction site (CCA**A**G to CC/A**G**G). No editing at site 5 prevented *MluI* restriction and therefore gave a full-length 311 bp product; editing at site 5 produced 2 bands (78 bp, 233 bp). No editing at site 6 produced 3 bands after *BstNI* digestion (22 bp, 81 bp, 208 bp; the 208 bp band was used as diagnostic for lack of editing), while editing followed by *BstNI* digestion produced 4 bands (22 bp, 69 bp, 81 bp, 139 bp; the 69 bp and 139 bp bands were treated as diagnostic for the presence of editing). Incomplete editing at either site manifested as a mix of full-length and cut products for each restriction.

**Figure 6:**
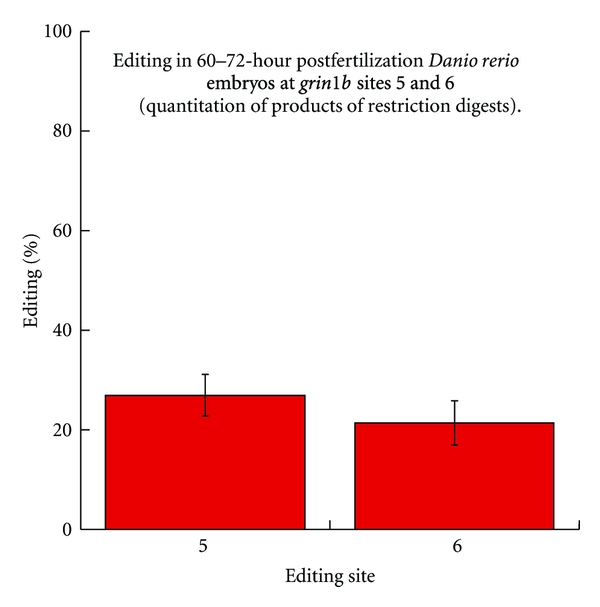
Editing frequencies and error bars (variation). Percent editing at sites 5 and 6 as measured by quantitation of the products of restriction digestion. Bars are standard deviation. Plotted with KaleidaGraph 4.

 Three identically primed (F3/R3; a 311 bp amplicon) PCRs from each of three independent, oligo dT-primed RTs (9 total amplifications) were used for these analyses: from each set of three reactions, one was used for *MluI* digestion, one for *BstNI*, and one for an untreated control. The products were extracted from an agarose gel and purified using a Qiagen gel extraction kit and protocol. Presence of the extracted band was confirmed via gel electrophoresis, and 25 uL restriction digests were performed as per NEB recommendations. Restriction digests were analyzed following gel electrophoresis using a Kodak Gel Logic imaging station and Kodak software. The intensity of the diagnostic versus uncut bands (also compared to unrestricted control bands) was analyzed and corrected for band size (as per [3]). The results confirmed approximate frequencies of editing that were initially predicted by sequence chromatograms (Figure 4) and integration of chromatographic signals (data not shown). Editing at site 5 was 26.98% with a standard deviation of 4.10%, and editing at site 6 was 21.36% with a standard deviation of 4.47% (Figure 6).

## 4. Discussion

 The positions edited occur within the reading frame of the gene at 3rd codon positions and do not result in amino acid substitutions. The positions of these edits indicate that they do not result in transcript recoding. This considerably complicates an analysis of the purpose of ADAR regulation at these loci; however, an intriguing possibility is that nonrecoding edits affect the binding of additional factors such as micro RNAs or positioning cues for ADARs. Searches of existing microRNA databases such as miRBase (http://www.mirbase.org/) for *D. rerio* miRNAs targeting the E5 and E6 region reveal that there are no known zebrafish miRNAs that bind in this area (within 100 bases 5′ of E6 and greater than 100 bases 3′ of E5), although we suggest the presence of additional unidentified novel miRNAs (or other small RNA species) could have an effect. A number of such possibilities for non-recoding edits are discussed by Morse and colleagues [4].

 There are several avenues of future research that may elucidate the function of non-recoding editing at these positions. Secondary structures of these regions were predicted using the mfold program developed by Zuker [5] in consideration of future structural confirmations (see [3]). Constructs with these changes can be made (separately or coordinately) with plasmid mutageneses and the affect on editing assayed in cell culture and transgenic animals. Examinations of binding affinities of zebrafish ADARs with edited versus unedited constructs can be pursued, as well as searches for differences in miRNA binding caused by these editing changes. This editing may also be coincidental, rather than functional, through mimicking the structure of editing enzyme substrates.

 The *grin1b* ortholog in humans is the *GRIN1* NMDA receptor gene. Several mutations in *GRIN1* are associated with severe mental retardation. When tested in *Xenopus* oocyte systems, increased calcium entry occurred with one mutated form [6] and the authors of this study point out the potential pathogenicity of the resultant increased calcium influx. Such results highlight the clinical value and human relevance of the study of *grin1b* in model vertebrates, such as Danio.

 RNA, in its many forms, plays a pivotal role in cellular processes. The processes of RNA editing and splicing along with micro RNAs, RNA interference, snRNAs, ribozymes, and so forth certainly point to RNA as a fundamental conductor and orchestrator of genetic instructions. Although RNA editing has been widely observed in many model organisms, finding a specific editing site is still a daunting task. We are interested in discovery of new editing sites as well as regulation of those sites, in a variety of model organisms. The Department of Biomolecular Sciences contains faculty who work collaboratively with a variety of experimental systems, and the record of Kung and colleagues' discovery of RNA editing in the *gria2* transcript of zebrafish [1] was a prompt and a clue as to what additional targets of editing might exist in transcripts from gene families in Danio. We were able to demonstrate the presence of two new sites of RNA editing in the *grin1b* gene transcript of zebrafish. We also call attention to the fact that site E6 was detectable only by direct sequencing and not by comparative methods; many editing sites, especially those that are unique to a single species, may remain to be discovered. Moreover, by characterizing editing in terms of frequency and location we hope to contribute to the current knowledge of RNA editing with the goal of participating in the full elucidation of this intriguing molecular process.

## Figures and Tables

**Table 1:** 

Predicted site	Exon	Location in transcript	Predicted recoding?
1	15	(Nucleotide 2630)	Yes (G**A**G/G**G**G) (E/G)
2	15	(Nucleotide 2659)	Yes (**A**GG/**G**GG) (R/G)
3	16	(Nucleotide 2690)	Yes (U**A**C/U**G**C) (Y/C)
4	17	(Nucleotide 2823)	No (AA**A**/AA**G**)
5*	17	(Nucleotide 2832)	No (GC**A**/GC**G**)
6* (see Figures 1(a) and 1(b))	16	(Nucleotide 2736)	No (CA**A**/CA**G**)

*Confirmed sites.

**Table 2:** 

Primer List.ZFGrin1b:	
F1 (CTGCGAAACCCATCAGATAAG)	
R1 (AACTCCAACACTGCTGAATC)	
F2 (AACTCCGGCATTGGAGAAGG)	
R2 (TTCACTGTGGCGTAGATGAAC)	
F3 (AATTTGGCAGCCTTCCTAGTG)	
R3 (AACAGCTCGCCCGTAGTAAC)	
E5.F1 (TATGATGTGGGGGTGGAGAC)	
E5.R1 (TGCCGATACCGAATCCAGAG)	
E6.F1 (GGAATTGCAGACACCAAACAC)	
E6.R1 (TGGCGGTACATGGTGCTAAG)	
